# Drowning during 2011-2017 in Langarud County 

**Published:** 2019-07

**Authors:** Faranak Sherafati, Ramin Gholampour Sigaroodi, Soheil Sirusbakht, Mehdi Saber Bazkiagoorabi

**Affiliations:** ^*a*^Healthcare Network of Langarud County, Guilan University of Medical Sciences, Guilan, Iran.

## Abstract

**Background:**

Many people die of drowning and its complications in different parts of the world annually. In Iran, drowning mostly occurs in summer. Langarud County in Guilan province is ranked third in terms of drowning incidence.

**Methods:**

The data of 44 drowned patients during years 2011-2017 were collected based on the age, sex, drowning place, living place, and the outcome of the drowning in Amini Hospital of Langerud county through completing questionnaires designed by the Ministry of Health, and were analyzed by SPSS software version19.

**Results:**

28.57% of the victims were aged between 10 and 20 years, and 26.5% were in the age group of 20-30 years old. 85.6% of the victims were male and 14.34% were female. 75.7% of drowned individuals were swimming outside the sea protected areas. 56.8% of the victims were tourists, and 79.5% of the drowning cases led to death.

**Conclusions:**

This study showed that drowning in Langarud County is an important issue beyond the province. For decreasing drowning cases, it is recommended to take the measures including making young and adolescent men aware of the dangers and reasons of drowning, youth swimming training, informing people of the risk of swimming outside the protected areas, and developing plans and improving rescue systems.

**Keywords:**

Drowning, Langarud County

